# Path Following Based on Waypoints and Real-Time Obstacle Avoidance Control of an Autonomous Underwater Vehicle

**DOI:** 10.3390/s20030795

**Published:** 2020-01-31

**Authors:** Xuliang Yao, Xiaowei Wang, Feng Wang, Le Zhang

**Affiliations:** 1 College of Automation, Harbin Engineering University, Harbin 150001, China; yaoxuliang@hrbeu.edu.cn (X.Y.); wangfeng3561@hrbeu.edu.cn (F.W.); 2 College of Mechanical engineering, Jiujiang Vocational and Technical College, Jiujiang 332007, China; 3 The Center for International Exchange and Cooperation, Jiujiang Vocational and Technical College, Jiujiang 332007, China; zhangle98538@163.com

**Keywords:** AUV, path following, obstacle avoidance, MPC, SMC

## Abstract

This paper studies three-dimensional (3D) straight line path following and obstacle avoidance control for an underactuated autonomous underwater vehicle (AUV) without lateral and vertical driving forces. Firstly, the expected angular velocities are designed by using two different methods in the kinematic controller. The first one is a traditional method based on Line-of-sight (LOS) guidance law, and the second one is an improved method based on model predictive control (MPC). At the same time, a penalty item is designed by using the obstacle information detected by onboard sensors, which can realize the real-time obstacle avoidance of the unknown obstacle. Then, in order to overcome the uncertainty of the dynamics model and the saturation of actual control input, the dynamic controller is designed by using sliding mode control (SMC) technology. Finally, in the simulation experiment, the performance of the improved control method is verified by comparison with two traditional control methods based on LOS guidance law. Since the constraint of an AUV’s angular velocities are considered in MPC, simulation results show that the improved control method uses MPC, and SMC not only improves the tracking quality of the AUV when switching paths near the waypoints and realizes real-time obstacle avoidance but also effectively reduces the mean square error (MSE) and saturation rate of the rudder angle. Therefore, this control method is more conducive to the system stability and saves energy.

## 1. Introduction

At present, autonomous underwater vehicles (AUVs) have gradually become an important tool in many fields such as ocean exploitation and scientific research. Because an AUV always follows the planned path during its mission, its path-following control is critical technology. When an AUV is operated in the open sea, its path is generally planned through a set of waypoints, with a straight line connecting every two adjacent waypoints. Its advantages are simple path planning and low computation. However, the controller design is not an easy task because the AUVs’ motions and model are coupled, nonlinear, and uncertain. For these reasons, the path-following control of the AUVs has been studied extensively worldwide [1].

Underactuated marine surface vessels and AUVs must calculate their ideal attitude in real time to realize path-following control. This task is accomplished by the kinematic controller according to the guidance law. In [2,3,4], the LOS guidance law was applied for path following. In order to improve the dynamic characteristics of tracking errors, the LOS guidance law with time-varying lookahead distance was presented in [5,6]. Alternatively, the vector field (VF) methodis also a popular guidance law [7,8]. Similar to the guidance problem, a lot of work has been done on the design of a dynamic controller in path following. The prevailing control methods for marine surface vessels and AUVs in path following include feedback linearizing control techniques [9,10,11], proportional-integral-derivative (PID) control [12,13], the backstepping method [14,15], Lyapunov direct method [16], robust control [17,18,19,20], adaptive control [21,22,23], gain scheduling control theory [24], sliding mode control (SMC) [25,26,27,28,29], neural network control [30], and fuzzy logic control [31], etc.

Generally, there are inherent physical constraints on the input of the control system, which will not only degrade the control performance but will also affect the stability of the system. None of the abovementioned papers has considered this problem. Model predictive control (MPC) is known as an optimization-based control method. MPC has a clear advantage to handle constraints, which represents the trend of the control of constrained systems. In [32], the constraint of the input was considered, and the LOS guidance law was optimized by using MPC algorithm to improve the waypoint tracking quality of an underactuated ship. In [33], the velocity and amplitude constraints of the rudder were considered, and the AUV’s attitude and depth control were optimized by using MPC algorithm. The limitation of the aforementioned papers is that the controllers are based on a nominal model. The principle of MPC is that it predicts the future state and output by using the current state and prediction model. Therefore, the accuracy of the prediction model has a great influence on the performance of MPC. Since the hydrodynamic parameters of the AUVs are uncertain and the motions of each degree of freedom are coupled and nonlinear, it is difficult to obtain the exact dynamic model of the AUVs. Therefore, using a nominal model to design MPC is difficult to guarantee the robustness of the system.

Straight-line path following based on waypoints is convenient for application, but the path is not smooth at each waypoint. The rudder angle is easily saturated when the path is switched near the waypoints during the AUV tracking of the path. Frequent saturation of the rudder angle will not only affect the stability of the system but will also increase the resistance and energy consumption of the AUV. Reference [34] applies the backstepping method to design the ship’s path-tracking controller and considers the system stability when the rudder angle is saturated. However, the saturation of the rudder angle does not fundamentally be alleviated in [34]. In addition, obstacle avoidance is often needed in path following. An obstacle avoidance guidance law is designed in [35]. However, this method needs to obtain the global information of obstacles in real-time, and it has high requirements for sensor configuration. In [36,37], based on the obstacle information detected by the onboard sensors, a spline curve is applied to replan the original path near the obstacle, and real-time obstacle avoidance is realized. However, replanning the path in real time greatly increases the computational burden of the controller.

In order to reduce the computation and simplify the structure of the controller, this paper designs the kinematics and dynamics controllers by using cascade control strategy, thereby providing a solution for the 3D straight line path following and obstacle avoidance of the AUVs. In the design of the kinematics controller, an improved control method is proposed by using MPC. In addition, in order to realize real-time obstacle avoidance, the penalty item for obstacle avoidance is designed according to the obstacle information detected by the onboard sensors. The optimal expected angular velocity satisfying the constraint can be obtained by using the improved control method, which can not only reduce the MSE and saturation rate of the rudder angle but also realize real-time obstacle avoidance. In the design of the dynamics controller, the saturation of control input is considered. The actual control signal is designed by SMC to control the expected speed of the AUV, which can not only overcome the uncertainty of the dynamics model but also ensure the stability of the system.

The remaining four sections of this paper are about the analysis of the control problem, the design of the kinematics and dynamics controller, the results and analysis of the simulation experiment, and the conclusion.

## 2. Analysis of the Control Problem

The underactuated AUV studied in this paper is not equipped with lateral and vertical driving forces but is equipped at the tail with a propeller to control the longitudinal speed, a pair of vertical rudders to control the yaw, and a pair of horizontal rudders to control the pitch. This is a very typical configuration of an AUV, which has the advantages of simplifying its mechanical structure, reducing cost, and improving reliability.

### 2.1. Kinematics and Dynamics Modeling

**Figure 1:**
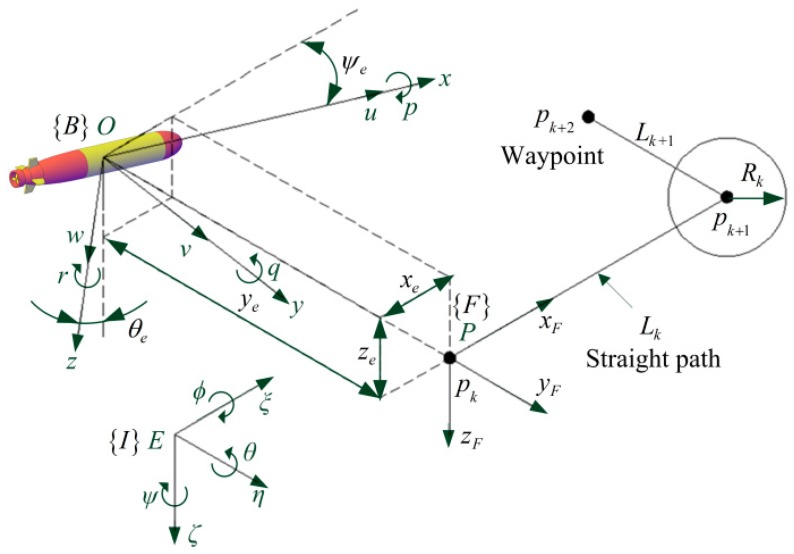
The control task of waypoint tracking is to steer the vehicle to follow the straight path between two adjacent waypoints. The straight path is switched to the next one when the AUV enters a circle of acceptance. The center of the circle is at $p_{k+1}$, and the radius is $R_{k}$.

As shown in Figure 1, in order to establish the six degrees of freedom (DOF) motion model of the AUV, the fixed coordinate system {*I*}:*E*−*ξηζ* and moving coordinate system {*B*}:*O*−*xyz* are introduced. AUV’s gravity and buoyancy are equal. The center of gravity (*CG*) is directly below the center of buoyancy (*CB*), which will generate rolling and pitching restoring moments to ensure the stability of the AUV. The distance between the *CG* and the *CB* (metacentric height) is $z_{g}$. The origin of {*I*} coordinate system is defined as a fixed point with the *ξ*-axis pointing north, the *η*-axis pointing east, and the *ζ*-axis pointing down. The origin of the {*B*} coordinate system is defined at the *CB*, with the *x*-axis pointing in front of the AUV, the *y*-axis pointing to the right, and the *z*-axis pointing down. Since the roll of the AUV is very small and does not need to be controlled, the kinematics and dynamics model of the AUV can be simplified to the following five DOFs after the roll is ignored:

(1) $$\dot{\eta }=J\left(\eta \right)v,$$

(2) $$M\dot{v}+C\left(v\right)v+D\left(v\right)v+g\left(\eta \right)=\tau +b,$$

where $\eta ={\left[\begin{array}{ccccc} \xi & \eta & \zeta & \theta & \psi \end{array}\right]}^{T}$. (ξ,η,ζ) represents the coordinates of *CB* in {*I*}, that is, the position of the AUV. θ and ψ represent the orientation of the AUV in {*I*}, that is, the pitching angle and yaw angle, respectively. J(η) represents the coordinate transformation from {*B*} to {*I*},

(3) $$J\left(\eta \right)=[\begin{array}{cc} J_{1}\left(\eta \right) & 0_{3\times 2}\\ 0_{2\times 3} & J_{2}\left(\eta \right) \end{array}],J_{1}\left(\eta \right)=\left[\begin{array}{ccc} \cos\psi \cos\theta & -\sin\psi & \cos\psi \sin\theta \\ \sin\psi \cos\theta & \cos\psi & \sin\psi \sin\theta \\ -\sin\theta & 0 & \cos\theta \end{array}\right],J_{2}\left(\eta \right)=\left[\begin{array}{cc} 1 & 0\\ 0 & \frac{1}{cos\theta } \end{array}\right].$$

$v={\left[\begin{array}{ccccc} u & v & w & q & r \end{array}\right]}^{T}$ denotes the velocities of the AUV defined in {*B*}. u,v,w are displacement velocities, that is, the respective surge, sway and heave velocities. q and r are angular velocities, that is, the pitch and yaw rates. The system matrices M,C(v),D(v) satisfy the properties $M=M^{T}$, $C\left(v\right)=-C{\left(v\right)}^{T},D\left(v\right)>0$. The restoring moments are defined as $g\left(\eta \right)={\left[\begin{array}{ccccc} 0 & 0 & 0 & M_{HS} & 0 \end{array}\right]}^{T}$, where $M_{HS}=-z_{g}G\sin\theta$ is the pitch restoring moment, and G is the gravity of the AUV. $\tau ={\left[\begin{array}{ccccc} X_{prop} & 0 & 0 & M_{fin} & N_{fin} \end{array}\right]}^{T}$ is the control vector, where $X_{prop},M_{fin},N_{fin}$ are the thrust and torque produced by the propeller and rudder. Vector b describes the model uncertainties. In order to make it convenient for the dynamic controller design, dynamics model (2) can be simplified as follows:

(4) $$\begin{array}{l} \dot{u}=\frac{1}{m_{11}}\left(m_{22}vr-m_{33}wq+d_{11}u+X_{prop}+b_{u}\right),\\ \dot{v}=\frac{1}{m_{22}}\left(-m_{11}ur+d_{22}v+b_{v}\right),\\ \dot{w}=\frac{1}{m_{33}}\left(m_{11}uq+d_{33}w+b_{w}\right),\\ \dot{q}=\frac{1}{m_{55}}\left[\left(m_{33}-m_{11}\right)uw+d_{55}q+M_{HS}+M_{fin}+b_{q}\right],\\ \dot{r}=\frac{1}{m_{66}}\left[\left(m_{11}-m_{22}\right)uv+d_{66}r+N_{fin}+b_{r}\right], \end{array}$$

where $m_{11}=m-X_{\dot{u}},m_{22}=m-Y_{\dot{v}},m_{33}=m-Z_{\dot{w}},m_{55}=I_{y}-M_{\dot{q}},m_{66}=I_{z}-N_{\dot{r}},d_{11}=X_{u}+X_{\left|u\right|u}\left|u\right|,d_{22}=Y_{v}+Y_{\left|v\right|v}\left|v\right|,$ $d_{33}=Z_{w}+Z_{\left|w\right|w}\left|w\right|,d_{55}=M_{q}+M_{\left|q\right|q}\left|q\right|,d_{66}=N_{r}+N_{\left|r\right|r}\left|r\right|.$ The symbol m denotes the mass of the AUV. $I_{y}$ and $I_{z}$ denote the moment of inertia. $b_{i}\left(i=u,v,w,q,r\right)$ represents model uncertainties. The remaining parameters are hydrodynamic coefficients.

### 2.2. Error Model

As shown in Figure 1, the routes are described in terms of waypoints which are fixed points in {*I*} frame. The reference path is the straight line between two adjacent waypoints. In order to establish the error model of the straight-line path followed, the path coordinate system $\left\{F\right\}:P-x_{F}y_{F}z_{F}$ is introduced. The current straight path $L_{k}$ starting at $p_{k}$ and ending at $p_{k+1}$ is defined. Here, the subscript *k* represents the sequence number of the straight path or waypoints.$\left(\xi_{k},\eta_{k},\zeta_{k}\right)$ represent coordinates of $p_{k}$, and $\left(\xi_{k+1},\eta_{k+1},\zeta_{k+1}\right)$ coordinates of $p_{k+1}$. The origin of {F} is placed at $p_{k}$ with the $x_{F}$-axis pointing towards $p_{k+1}$, the $y_{F}$-axis pointing to the right, and the $z_{F}$-axis pointing down. The $y_{F}$ and $z_{F}$ coordinates of the AUV in the {F} frame equal the lateral error and vertical error, respectively. The orientation of {*B*} in {*I*} is defined as $A_{B}={\left[\begin{array}{cc} \theta & \psi \end{array}\right]}^{T}$, and the orientation of {*F*} in {*I*} as $A_{F}={\left[\begin{array}{cc} \theta_{F} & \psi_{F} \end{array}\right]}^{T}$. $\theta_{F}$ and $\psi_{F}$ are calculated as follows:

(5) $$\begin{array}{l} \theta_{F}=-\arctan2\left(\Delta \zeta ,\sqrt{{\left(\Delta \xi \right)}^{2}+{\left(\Delta \eta \right)}^{2}}\right),\\ \psi_{F}=\arctan2\left(\Delta \eta ,\Delta \xi \right), \end{array}$$

where $\Delta \xi =\xi_{k+1}-\xi_{k},\Delta \eta =\eta_{k+1}-\eta_{k},\Delta \zeta =\zeta_{k+1}-\zeta_{k}$.

The orientation of {*B*} relative to the {*F*} frame is defined $A_{e}={\left[\begin{array}{cc} \theta_{e} & \psi_{e} \end{array}\right]}^{T}$, then

(6) $$A_{e}=\left[\begin{array}{c} \theta_{e}\\ \psi_{e} \end{array}\right]=\left[\begin{array}{cc} 1 & 0\\ 0 & \cos\theta_{F} \end{array}\right]\left(A_{B}-A_{F}\right)$$

Since $A_{F}$ is constant in the current straight path, then the derivative of Equation (6) yields

(7) $${\dot{A}}_{e}=\left[\begin{array}{c} {\dot{\theta }}_{e}\\ {\dot{\psi }}_{e} \end{array}\right]=\left[\begin{array}{cc} 1 & 0\\ 0 & \cos\theta_{F} \end{array}\right]\left[\begin{array}{c} \dot{\theta }\\ \dot{\psi } \end{array}\right]=\left[\begin{array}{c} \dot{\theta }\\ \dot{\psi }\cos\theta_{F} \end{array}\right]=\left[\begin{array}{c} q\\ \frac{\cos\theta_{F}}{\cos\theta }r \end{array}\right].$$

The inertial coordinates of *O* in {*I*} frame are $P_{O}={\left[\begin{array}{ccc} \xi_{O} & \eta_{O} & \zeta_{O} \end{array}\right]}^{T}$, the inertial coordinates of *P* in {*I*} frame are $P_{P}={\left[\begin{array}{ccc} \xi_{k} & \eta_{k} & \zeta_{k} \end{array}\right]}^{T}$, and the coordinates of *O* in {*F*} frame are $P_{e}={\left[\begin{array}{ccc} x_{e} & y_{e} & z_{e} \end{array}\right]}^{T}$, then

(8) $$P_{e}={\left[\begin{array}{ccc} x_{e} & y_{e} & z_{e} \end{array}\right]}^{T}=R_{I}^{F}\left(P_{O}-P_{P}\right),\;R_{I}^{F}=\left[\begin{array}{ccc} \cos\psi_{F}\cos\theta_{F} & \sin\psi_{F}\cos\theta_{F} & -\sin\theta_{F}\\ -\sin\psi_{F} & \cos\psi_{F} & 0\\ \cos\psi_{F}\sin\theta_{F} & \sin\psi_{F}\sin\theta_{F} & \cos\theta_{F} \end{array}\right].$$

$R_{I}^{F}$ is the coordinate transformation from {*I*} to {*F*}.

Then, the derivative of Equation (8) yields

(9) $${\dot{P}}_{e}={\dot{R}}_{I}^{F}\left(P_{O}-P_{P}\right)+R_{I}^{F}\left({\dot{P}}_{O}-{\dot{P}}_{P}\right)=R_{I}^{F}{\dot{P}}_{O}=R_{I}^{F}J_{1}\left(\eta \right)\left[\begin{array}{c} u\\ v\\ w \end{array}\right]=\left[\begin{array}{c} u\cos\psi_{e}\cos\theta_{e}-v\sin\psi_{e}+w\cos\psi_{e}\sin\theta_{e}\\ u\sin\psi_{e}\cos\theta_{e}+v\cos\psi_{e}+w\sin\psi_{e}\sin\theta_{e}\\ -u\sin\theta_{e}+w\cos\theta_{e} \end{array}\right].$$

## 3. Controller Design

In this paper, the control objective is to make the AUV track the straight lines path between the waypoints at a constant speed $u_{d}>0$, which is more conducive to energy saving. In the kinematics controller, the expected angular velocities $q_{d}$ and $r_{d}$ are designed by using two different methods, and then the obstacle avoidance penalty is designed based on the obstacle information detected by onboard sensors, where $q_{d}$ and $r_{d}$ are the expected pitch rate and yaw rate, respectively. The expected velocities are developed as virtual control inputs, and they are fed to the dynamic controller. Then, the actual control input variables $n_{p},\delta_{s},\delta_{r}$ are derived in the dynamic controller by using sliding mode control (SMC) technology, where $n_{p},\delta_{s},\delta_{r}$ are the respective speed of the propeller, sternplane rudder angle, and vertical rudder angle.

### 3.1. Kinematics Controller

#### 3.1.1. Kinematics Controller Design Based on LOS Guidance Law

Since the along-track error $x_{e}$ does not need to be controlled, error model (9) can be simplified as follows:

(10) $$\left[\begin{array}{c} {\dot{\bar{y}}}_{e}\\ {\dot{\bar{z}}}_{e} \end{array}\right]=\left[\begin{array}{c} u\sin\psi_{e}\cos\theta_{e}+b_{y}\\ -u\sin\theta_{e}+b_{z} \end{array}\right],$$

where ${\bar{y}}_{e}=y_{e}-y_{ed}$, ${\bar{z}}_{e}=z_{e}-z_{ed}$. $y_{ed}$ and $z_{ed}$ are penalty items added for obstacle avoidance, which are set to zero when there are no obstacles. When there is an obstacle, setting $y_{ed}$ or $z_{ed}$ to a positive value can avoid the obstacle from the right or below, and vice versa. The calculation method of the penalty item will be introduced in detail later. Because the AUV studied in this paper lacks lateral and vertical driving forces, the displacement velocities *v* and *w* are very small. $b_{y}=v\cos\psi_{e}+w\sin\psi_{e}\sin\theta_{e},b_{z}=w\cos\theta_{e}$ are bounded. By applying the LOS guidance law, the expected values of $\theta_{e}$ and $\psi_{e}$ can be designed as follows:

(11) $$\begin{array}{l} \theta_{ed}=\arctan\left({\bar{z}}_{e}/\Delta_{\theta }\right),\\ \psi_{ed}=-\arctan\left({\bar{y}}_{e}/\Delta_{\psi }\right), \end{array}$$

where $\Delta_{\theta }>0$ and $\Delta_{\psi }>0$ are look-ahead distances.

After defining the error variable ${\tilde{\theta }}_{e}=\theta_{ed}-\theta_{e},{\tilde{\psi }}_{e}=\psi_{ed}-\psi_{e}$, the error model (10) can be rewritten as follows:

(12) $$\left[\begin{array}{c} {\dot{\bar{y}}}_{e}\\ {\dot{\bar{z}}}_{e} \end{array}\right]=A\left(t\right)\left[\begin{array}{c} {\bar{y}}_{e}\\ {\bar{z}}_{e} \end{array}\right]+B\left(t\right)\left[\begin{array}{c} {\tilde{\theta }}_{e}\\ {\tilde{\psi }}_{e} \end{array}\right]+\left[\begin{array}{c} b_{y}\\ b_{z} \end{array}\right],$$

where

$$\begin{array}{c} A\left(t\right)=\left[\begin{array}{cc} A_{11} & 0\\ 0 & A_{22} \end{array}\right],B\left(t\right)=\left[\begin{array}{cc} B_{11} & B_{12}\\ B_{21} & 0 \end{array}\right],\\ A_{11}=-\frac{u}{\sqrt{{\bar{y}}_{e}^{2}+\Delta_{\psi }^{2}}}\frac{\Delta_{\theta }}{\sqrt{{\bar{z}}_{e}^{2}+\Delta_{\theta }^{2}}},A_{22}=-\frac{u}{\sqrt{{\bar{z}}_{e}^{2}+\Delta_{\theta }^{2}}},B_{11}=u\sin\psi_{ed}\cos{\tilde{\psi }}_{e}\left[\frac{\left(\cos{\tilde{\theta }}_{e}-1\right)}{{\tilde{\theta }}_{e}}\cos\theta_{ed}+\frac{\sin{\tilde{\theta }}_{e}}{{\tilde{\theta }}_{e}}\sin\theta_{ed}\right],\\ B_{12}=u\left[\frac{\left(\cos{\tilde{\psi }}_{e}-1\right)}{{\tilde{\psi }}_{e}}\sin\psi_{ed}\cos\theta_{ed}-\frac{\sin{\tilde{\psi }}_{e}}{{\tilde{\psi }}_{e}}\cos\psi_{ed}\cos\theta_{e}\right],B_{21}=u\left[\frac{\sin{\tilde{\theta }}_{e}}{{\tilde{\theta }}_{e}}\cos\theta_{ed}-\frac{\left(\cos{\tilde{\theta }}_{e}-1\right)}{{\tilde{\theta }}_{e}}\sin\theta_{ed}\right]. \end{array}$$

Since A(t) is negative and $B\left(t\right),b_{y},b_{z}$ are bounded, according to the cascade system theory, the stability of the system (12) can be guaranteed as long as ${\tilde{\theta }}_{e}$ and ${\tilde{\psi }}_{e}$ are stable. Next, the virtual control input is designed through the backstepping method to ensure the stability of ${\tilde{\theta }}_{e}$ and ${\tilde{\psi }}_{e}$. The Lyapunov function is defined as follows:

(13) $$V_{1}=\frac{1}{2}{\tilde{\theta }}_{e}^{2}+\frac{1}{2}{\tilde{\psi }}_{e}^{2}.$$

The expected angular velocities can be designed as

(14) $$\begin{array}{l} q_{d}={\dot{\theta }}_{ed}+k_{q}{\tilde{\theta }}_{e},\\ r_{d}=\frac{\cos\theta }{\cos\theta_{F}}\left({\dot{\psi }}_{ed}+k_{r}{\tilde{\psi }}_{e}\right). \end{array}$$

Then, the derivative of $V_{1}$ yields ${\dot{V}}_{1}=-k_{q}{\tilde{\theta }}_{e}^{2}-k_{r}{\tilde{\psi }}_{e}^{2}\leq -2k_{V_{1}}V_{1}\leq 0$, where $k_{q}>0,k_{r}>0,k_{V_{1}}=\min\left[k_{q},k_{r}\right]$. Although the above traditional control method based on the LOS guidance law is easy to calculate and apply, it still has some shortcomings. When $\Delta_{\theta }$ and $\Delta_{\psi }$ are set to be small, the path tracking is prone to overshoot, otherwise the tracking errors converge slowly. When $k_{q}$ and $k_{r}$ are set to be small, the path tracking quality will decrease, otherwise the rudder angle is prone to saturation. The reason is that the expected angular velocities obtained by the above traditional control method are not the optimal value and do not meet the constraint conditions. In order to improve the control quality, the next step is to use MPC to redesign the kinematic controller.

#### 3.1.2. Kinematics Controller Design Based on MPC

The response of the dynamic controller to the expected angular velocity can be described as follows:

(15) $$\begin{array}{l} \dot{q}=\frac{1}{T_{1}}\left(q_{d}-q\right),\\ \dot{r}=\frac{1}{T_{2}}\left(r_{d}-r\right), \end{array}$$

where $T_{1}>0$ and $T_{2}>0$ are adjustable time constants.

##### The Predictive Model

Because the AUV studied in this paper lacks lateral and vertical driving forces, the displacement velocities *v* and *w* are very small, which can be ignored to simplify the controller. Since along-track error *x_e_* does not need to be controlled, according to Equations (7), (9), and (15), the control objective can be simplified to the stabilization problem as follows:

(16) $$\left[\begin{array}{c} {\dot{\bar{y}}}_{e}\\ {\dot{\bar{z}}}_{e}\\ {\dot{\theta }}_{e}\\ {\dot{\psi }}_{e}\\ \dot{q}\\ \dot{r} \end{array}\right]=\left[\begin{array}{c} u\sin\psi_{e}\cos\theta_{e}\\ -u\sin\theta_{e}\\ q\\ \frac{\cos\theta_{F}}{\cos\theta }r\\ \frac{1}{T_{1}}\left(q_{d}-q\right)\\ \frac{1}{T_{2}}{\left(r_{d}-r\right)}_{2} \end{array}\right]=\left[\begin{array}{c} uk_{y}\psi_{e}\\ -uk_{z}\theta_{e}\\ q\\ \frac{\cos\theta_{F}}{\cos\theta }r\\ \frac{1}{T_{1}}\left(q_{d}-q\right)\\ \frac{1}{T_{2}}\left(r_{d}-r\right) \end{array}\right],$$

where $k_{y}=\cos\theta_{e}\sin\psi_{e}/\psi_{e},k_{z}=\sin\theta_{e}/\theta_{e}$. In order to avoid singularities, $k_{y}$ is set to $k_{y}=\cos\theta_{e}$ when $\left|\psi_{e}\right|\leq \pi /12$ and $k_{z}$ is set to $k_{z}=1$ when $\left|\theta_{e}\right|\leq \pi /12$. The system (16) can be rewritten as

(17) $$\dot{x}=f\left(x,u\right),$$

where $x={\left[\begin{array}{llllll} {\bar{y}}_{e} & {\bar{z}}_{e} & \theta_{e} & \psi_{e} & q & r \end{array}\right]}^{T}$, $u={\left[\begin{array}{cc} q_{d} & r_{d} \end{array}\right]}^{T}$. Obviously, the equilibrium points of system (17) are ***x*** = 0 and ***u*** = 0. By discretizing system (17), the following can be obtained:

(18) $$\begin{array}{l} x_{k+1,k}=A_{k}x_{k,k}+B_{k}u_{k,k},\\ y_{k,k}=C_{k}x_{k,k}, \end{array}$$

where

$$A_{k}=\left[\begin{array}{cccccc} 1 & 0 & 0 & Tuk_{y} & 0 & 0\\ 0 & 1 & -Tuk_{z} & 0 & 0 & 0\\ 0 & 0 & 1 & 0 & T & 0\\ 0 & 0 & 0 & 1 & 0 & \frac{T\cos\theta_{F}}{\cos\theta }\\ 0 & 0 & 0 & 0 & 1-\frac{T}{T_{1}} & 0\\ 0 & 0 & 0 & 0 & 0 & 1-\frac{T}{T_{2}} \end{array}\right],B_{k}=\left[\begin{array}{cc} 0 & 0\\ 0 & 0\\ 0 & 0\\ 0 & 0\\ \frac{T}{T_{1}} & 0\\ 0 & \frac{T}{T_{2}} \end{array}\right],C_{k}=I_{6}.$$

T is the sampling time. Here the subscript *k* represents the sampling time series. Using (18), the future system state can be predicted as

(19) $$\begin{array}{ccc} x_{k+1,k} & = & A_{k}x_{k,k}+B_{k}u_{k,k},\\ x_{k+2,k} & = & A_{k}x_{k+1,k}+B_{k}u_{k+1,k}=A_{k}^{2}x_{k,k}+A_{k}B_{k}u_{k,k}+B_{k}u_{k+1,k},\\ x_{k+3,k} & = & A_{k}x_{k+2,k}+B_{k}u_{k+2,k}=A_{k}^{3}x_{k,k}+A_{k}^{2}B_{k}u_{k,k}+A_{k}B_{k}u_{k+1,k}+B_{k}u_{k+2,k},\\ & \vdots & \\ x_{k+N_{p},k} & = & A_{k}^{N_{p}}x_{k,k}+A_{k}^{N_{p}-1}B_{k}u_{k,k}+\cdots +A_{k}^{N_{p}-N_{c}}B_{k}u_{k+N_{c}-1,k}, \end{array}$$

where $N_{c}\leq N_{p}$, $N_{c}$ is the control horizon, $N_{p}$ is the prediction horizon. Then, the future system output can be predicted as

(20) $$\begin{array}{ccc} y_{k+1,k} & = & C_{k}A_{k}x_{k,k}+C_{k}B_{k}u_{k,k},\\ y_{k+2,k} & = & C_{k}A_{k}^{2}x_{k,k}+C_{k}A_{k}B_{k}u_{k,k}+C_{k}B_{k}u_{k+1,k},\\ y_{k+3,k} & = & C_{k}A_{k}^{3}x_{k,k}+C_{k}A_{k}^{2}B_{k}u_{k,k}+C_{k}A_{k}B_{k}u_{k+1,k}+C_{k}B_{k}u_{k+2,k},\\ & \vdots & \\ y_{k+N_{p},k} & = & C_{k}A_{k}^{N_{p}}x_{k,k}+C_{k}A_{k}^{N_{p}-1}B_{k}u_{k,k}+\cdots +C_{k}A_{k}^{N_{p}-N_{c}}B_{k}u_{k+N_{c}-1,k}. \end{array}$$

The future system output can be rewritten as

(21) $$Y_{k+1,k}=\Psi x_{k,k}+\Theta U_{k,k},$$

where

$$Y_{k+1,k}=\left[\begin{array}{c} y_{k+1,k}\\ y_{k+2,k}\\ y_{k+3,k}\\ \vdots \\ y_{k+N_{p},k} \end{array}\right],U_{k,k}=\left[\begin{array}{c} u_{k,k}\\ u_{k+1,k}\\ u_{k+2,k}\\ \vdots \\ u_{k+N_{c}-1,k} \end{array}\right],\Psi =\left[\begin{array}{c} C_{k}A_{k}\\ C_{k}A_{k}^{2}\\ C_{k}A_{k}^{3}\\ \vdots \\ C_{k}A_{k}^{N_{p}} \end{array}\right],\Theta =\left[\begin{array}{ccccc} C_{k}B_{k} & 0 & 0 & \cdots & 0\\ C_{k}A_{k}B_{k} & C_{k}B_{k} & 0 & \cdots & 0\\ C_{k}A_{k}^{2}B_{k} & C_{k}A_{k}B_{k} & C_{k}B_{k} & \cdots & 0\\ \vdots & \vdots & \vdots & \ddots & \vdots \\ C_{k}A_{k}^{N_{p}-1}B_{k} & C_{k}A_{k}^{N_{p}-2}B_{k} & C_{k}A_{k}^{N_{p}-3}B_{k} & \cdots & C_{k}A_{k}^{N_{p}-N_{c}}B_{k} \end{array}\right].$$

##### The Control Constraint

In the actual control system, due to the saturation limitation of the rudder angle, the AUV’s angular velocity is also constrained. Here, the control constraints are considered as

(22) $$u_{\min}\leq u_{k+t,k}\leq u_{\max},t=0,1,\cdots N_{c}-1.$$

The control constraints (22) can be translated into linear inequalities as follows:

(23) $$\left[\begin{array}{r} M_{c}\\ -M_{c} \end{array}\right]U_{k,k}\leq \left[\begin{array}{c} N_{\max}\\ N_{\min} \end{array}\right],$$

where $M_{c}=I_{N_{c}}⊗I_{2},N_{\max}=1_{N_{c}}⊗u_{\max},N_{\min}=-1_{N_{c}}⊗u_{\min},1_{N_{c}}^{T}={\left[\begin{array}{ccccc} 1 & 1 & 1 & \cdots & 1 \end{array}\right]}_{1\times N_{c}}.$ The symbol ⊗ is Kronecker product.

##### Optimization with Control Constraint

For system (18), the task of MPC can be equivalent to calculating the optimal control input $U_{k,k}$ at each sampling time k to minimize cost functionas follows:

(24) $$J_{k,k}=Y_{k+1,k}^{T}\bar{Q}Y_{k+1,k}+U_{k,k}^{T}\bar{R}U_{k,k},$$

where $\bar{Q}=I_{N_{p}}⊗Q$, $\bar{R}=I_{N_{c}}⊗R$, $Q=diag\left(Q_{11},Q_{22},Q_{33},Q_{44},Q_{55},Q_{66}\right)$, $R=diag\left(R_{11},R_{22}\right)$ are positive definite weighting matrices. The next step is to design constraints to ensure the stability of MPC.

The cost function at sampling time k−1 is defined as

(25) $$J_{k-1,k-1}=Y_{k,k-1}^{T}\bar{Q}Y_{k,k-1}+U_{k-1,k-1}^{T}\bar{R}U_{k-1,k-1},$$

where

$$U_{k-1,k-1}=\left[\begin{array}{c} u_{k-1,k-1}\\ u_{k,k-1}\\ u_{k+1,k-1}\\ \vdots \\ u_{k+N_{c}-2,k-1} \end{array}\right],Y_{k,k-1}=\left[\begin{array}{c} y_{k,k-1}\\ y_{k+1,k-1}\\ y_{k+2,k-1}\\ \vdots \\ y_{k+N_{p}-1,k-1} \end{array}\right].$$

A feasible control variable at time k is defined, and the predicted output variable is

(26) $${\bar{U}}_{k,k}=\left[\begin{array}{c} u_{k,k-1}\\ u_{k+1,k-1}\\ \vdots \\ u_{k+N_{c}-2,k-1}\\ u_{k+N_{c}-1,k} \end{array}\right],{\bar{Y}}_{k+1,k}=\left[\begin{array}{c} y_{k+1,k-1}\\ y_{k+2,k-1}\\ \vdots \\ y_{k+N_{p}-1,k-1}\\ {\bar{y}}_{k+N_{p},k} \end{array}\right].$$

Define $\Delta J_{k,k}=J_{k,k}-J_{k-1,k-1}$, then

$$\begin{array}{ll} \Delta J_{k,k} & =J_{k,k}-J_{k-1,k-1},\\ & =Y_{k+1,k}^{T}\bar{Q}Y_{k+1,k}+U_{k,k}^{T}\bar{R}U_{k,k}-Y_{k,k-1}^{T}\bar{Q}Y_{k,k-1}-U_{k-1,k-1}^{T}\bar{R}U_{k-1,k-1},\\ & \leq {\bar{Y}}_{k+1,k}^{T}\bar{Q}{\bar{Y}}_{k+1,k}+{\bar{U}}_{k,k}^{T}\bar{R}{\bar{U}}_{k,k}-Y_{k,k-1}^{T}\bar{Q}Y_{k,k-1}-U_{k-1,k-1}^{T}\bar{R}U_{k-1,k-1},\\ & \leq {\bar{y}}_{k+N_{p},k}^{T}Q{\bar{y}}_{k+N_{p},k}-y_{k,k-1}^{T}Qy_{k,k-1}+u_{k+N_{c}-1,k}^{T}Ru_{k+N_{c}-1,k}-u_{k-1,k-1}^{T}Ru_{k-1,k-1}. \end{array}$$

The stability of MPC can be guaranteed as long as the following constraints are met,

(27) $${\bar{y}}_{k+N_{p},k}^{T}Q{\bar{y}}_{k+N_{p},k}-y_{k,k}^{T}Qy_{k,k}+u_{k+N_{c}-1,k}^{T}Ru_{k+N_{c}-1,k}-u_{k-1,k-1}^{T}Ru_{k-1,k-1}\leq 0,$$

where

$$\begin{array}{l} {\bar{y}}_{k+N_{p},k}^{T}=\bar{\Psi }x_{k,k}+{\bar{\Theta }}_{1}C_{1}U_{k-1,k-1}+{\bar{\Theta }}_{2}C_{2}U_{k,k},u_{k+N_{c}-1,k}=C_{2}U_{k,k},u_{k-1,k-1}=C_{3}U_{k-1,k-1},\\ \bar{\Psi }=C_{k}A_{k}^{N_{p}},{\bar{\Theta }}_{1}=C_{k}\left[\begin{array}{ccccc} A_{k}^{N_{p}-1}B_{k} & A_{k}^{N_{p}-2}B_{k} & A_{k}^{N_{p}-3}B_{k} & \cdots & A_{k}^{N_{p}-N_{c}+1}B_{k} \end{array}\right],{\bar{\Theta }}_{2}=C_{k}A_{k}^{N_{p}-N_{c}}B_{k},\\ C_{1}=\left[\begin{array}{cc} 0_{\left(N_{c}-1\right)\times 1} & I_{N_{c}-1} \end{array}\right]⊗I_{2},C_{2}=\left[\begin{array}{cc} 0_{1\times \left(N_{c}-1\right)} & 1 \end{array}\right]⊗I_{2},C_{3}=\left[\begin{array}{cc} 1 & 0_{1\times \left(N_{c}-1\right)} \end{array}\right]⊗I_{2}. \end{array}$$

The constrained variables are parameterized by parameter vector $U_{k,k}$, making it convenient to solve the optimization problem. By inserting Equation (21) into Equation (24), $J_{k,k}$ is expressed as

(28) $$J_{k,k}={\left[\Psi x_{k,k}\right]}^{T}\bar{Q}\Psi x_{k,k}+U_{k,k}^{T}EU_{k,k}+2U_{k,k}^{T}F,$$

where $E=\Theta^{T}\bar{Q}\Theta +\bar{R},F=\Theta^{T}\bar{Q}\Psi x_{k,k}$. Because ${\left[\Psi x_{k,k}\right]}^{T}\bar{Q}\Psi x_{k,k}$ is constant, the solving of optimization problems under the constraints at every sampling time is rewritten as the following QP problem:

(29) $$\begin{array}{l} \underset{U_{k,k}}{\min}J_{k,k}=U_{k,k}^{T}EU_{k,k}+2U_{k,k}^{T}F,\\ s.t.\left[\begin{array}{r} M_{c}\\ -M_{c} \end{array}\right]U_{k,k}\leq \left[\begin{array}{c} N_{\max}\\ N_{\min} \end{array}\right],\\ \begin{array}{cc} & {\bar{y}}_{k+N_{p},k}^{T}Q{\bar{y}}_{k+N_{p},k}-y_{k,k}^{T}Qy_{k,k}+u_{k+N_{c}-1,k}^{T}Ru_{k+N_{c}-1,k}-u_{k-1,k-1}^{T}Ru_{k-1,k-1}\leq 0 \end{array}. \end{array}$$

At each sampling time, the optimal control input $U_{k,k}$ can be obtained by solving Equation (29). Then, the first element $u_{k,k}$ is implemented as the optimal expected value of the angular velocity as follows:

(30) $${\left[\begin{array}{c} q_{d}\\ r_{d} \end{array}\right]}_{k,k}=u_{k,k}.$$

By repeating the above calculation process, the optimal expected angular velocity at each sampling moment can be obtained in real time.

#### 3.1.3. Obstacle Detection and Calculation of the Penalty Term for Obstacle Avoidance

**Figure 2:**
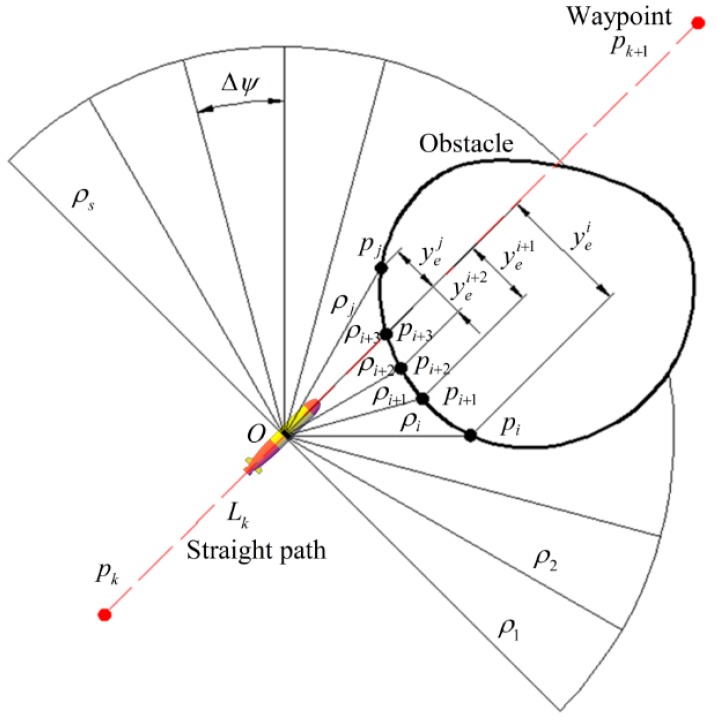
The obstacle avoidance diagram.

Nowadays, several types of sensors can be used to detect obstacles. For example, in [38,39], light detection and ranging (LiDAR) sensors or stereoscopic cameras are used in obstacle avoidance control of robots. In [37,40], sonar is used as the sensor to detect obstacles for obstacle avoidance control of underwater robots. In this paper, the AUV is configured with sonaras shown in Figure 2, which can obtain the information of obstacles in real time. The detection range, angle of view, detection period, and beam number of the sonar are 100 m, 180°, 0.5 s, and 61, respectively. The angle between any two beams of the sonar is Δψ. The sensor can return a set of data $\rho =\left[\rho_{1},\rho_{2},\rho_{i},\cdots \rho_{s}\right]$ in real time where $\rho_{i},\left(i=1\cdots s\right)$ represents the distance between AUV and the obstacle in the direction of the *i*th beam of sonar, that is, the distance between ***O*** and the intersection point $p_{i}$. If there are no obstacles, then $\rho_{i}=0,\left(i=1\cdots s\right)$. If there is a set of continuous non-zero data $\left[\rho_{i},\cdots \rho_{j}\right],\left(1\leq i\leq j\leq s\right)$ in the received data ***ρ***, it means there is an obstacle ahead. At this point, it is necessary to calculate the penalty term in real time to avoid the obstacle.

Since the AUV sails in the horizontal plane for most of the time, the obstacle avoidance in the horizontal plane is taken as an example to introduce the calculation procedure of the penalty item.

Step 1: The coordinates of intersection points $p_{c},\left(c=i\cdots j\right)$ in a fixed coordinate system are calculated according to the following equations:
  (31) $$\begin{array}{l} \left[\begin{array}{c} \xi_{c}\\ \eta_{c} \end{array}\right]=\left[\begin{array}{cc} \cos\psi_{c} & -\sin\psi_{c}\\ \sin\psi_{c} & \cos\psi_{c} \end{array}\right]\left[\begin{array}{c} \rho_{c}\\ 0 \end{array}\right]+\left[\begin{array}{c} \xi \\ \eta \end{array}\right],\\ \psi_{c}=\psi +\left(\frac{s+1}{2}-c\right)\Delta \psi ,\left(c=i,\cdots j\right). \end{array}$$
Step 2: The distance between the intersection point $p_{c},\left(c=i\cdots j\right)$ and the current path is calculated according to the following equation:
  (32) $$y_{e}^{c}=\left[\begin{array}{cc} 0 & 1 \end{array}\right]\left[\begin{array}{cc} \cos\psi_{F} & \sin\psi_{F}\\ -\sin\psi_{F} & \cos\psi_{F} \end{array}\right]\left(\left[\begin{array}{c} \xi_{c}\\ \eta_{c} \end{array}\right]-\left[\begin{array}{c} \xi_{k}\\ \eta_{k} \end{array}\right]\right),\left(c=i,\cdots j\right).$$
Step 3: If all the data $y_{e}^{c},\left(c=i,\cdots j\right)$ are greater than zero, then the obstacle is on the right side of the path. At this point, if $\min\left(y_{e}^{c}\right)>y_{e}^{s},\left(c=i,\cdots j\right)$ is satisfied, then set $y_{ed}=0$. Otherwise, choose to avoid the obstacle from the left side of the obstacle and set $y_{ed}=\min\left(y_{e}^{c}\right)-y_{e}^{s}$ until all the received data ***ρ*** values are zero, then set $y_{ed}=0$, where $y_{e}^{s}>0$ is the safe distance between the AUV and an obstacle.
Step 4: If all the data $y_{e}^{c},\left(c=i,\cdots j\right)$ are less than zero, then the obstacle is on the left side of the path. At this point, if $\min\left(\left|y_{e}^{c}\right|\right)>y_{e}^{s},\left(c=i,\cdots j\right)$ is satisfied, then set $y_{ed}=0$. Otherwise, choose to avoid the obstacle from the right side of the obstacle and set $y_{ed}=\max\left(y_{e}^{c}\right)+y_{e}^{s}$ until all the received data ***ρ*** values are zero, then set $y_{ed}=0$.
Step 5: If $\min\left(y_{e}^{c}\right)<0,\max\left(y_{e}^{c}\right)>0,\left(c=i,\cdots j\right)$ is satisfied, then the obstacle is on the path ahead. At this point, if $\left|\min\left(y_{e}^{c}\right)\right|<\left|\max\left(y_{e}^{c}\right)\right|,\left(c=i,\cdots j\right)$ is satisfied, then choose to avoid the obstacle from the left side of the obstacle and set $y_{ed}=\min\left(y_{e}^{c}\right)-y_{e}^{s}$. Otherwise, choose to avoid the obstacle from the right side of the obstacle and set $y_{ed}=\max\left(y_{e}^{c}\right)+y_{e}^{s}$ until all the received data ***ρ*** values are zero, then set $y_{ed}=0$.

### 3.2. Dynamics Controller

Next, the design of the dynamic controller is introduced to realize the tracking control of the expected velocity signal. Since the underactuated AUV lacks lateral and vertical thrusters, dynamic Equation (4) can be simplified as follows:

(33) $${\dot{v}}_{a}=C_{a}v_{a}+G_{a}+D_{a}{\bar{\tau }}_{a}+b_{a},$$

where

$$v_{a}=\left[\begin{array}{c} u\\ q\\ r \end{array}\right],C_{a}=\left[\begin{array}{ccc} \frac{d_{11}}{m_{11}} & -\frac{m_{33}}{m_{11}}w & \frac{m_{22}}{m_{11}}v\\ \frac{m_{33}-m_{11}}{m_{55}}w & \frac{d_{55}}{m_{55}} & 0\\ \frac{m_{11}-m_{22}}{m_{66}}v & 0 & \frac{d_{66}}{m_{66}} \end{array}\right],G_{a}=\left[\begin{array}{c} 0\\ \frac{M_{HS}}{m_{55}}\\ 0 \end{array}\right],D_{a}=\left[\begin{array}{ccc} \frac{k_{p}}{m_{11}} & 0 & 0\\ 0 & \frac{k_{\delta }}{m_{55}} & 0\\ 0 & 0 & \frac{k_{\delta }}{m_{66}} \end{array}\right],{\bar{\tau }}_{a}=\left[\begin{array}{c} {\bar{n}}_{p}^{2}\\ {\bar{\delta }}_{s}\\ {\bar{\delta }}_{r} \end{array}\right],b_{a}=\left[\begin{array}{c} b_{u}\\ b_{q}\\ b_{r} \end{array}\right].$$

$k_{\delta }$,$k_{p}$ are the lift coefficient of the rudder and thrust coefficient of the propeller, respectively.

The actual control inputs have saturation limits, and the saturation value of the control input is defined as follows:

(34) $$\Delta \tau_{a}={\bar{\tau }}_{a}-\tau_{a},$$

where $\tau_{a}={\left[\begin{array}{ccc} n_{p}^{2} & \delta_{s} & \delta_{r} \end{array}\right]}^{T}$.

The following auxiliary systems are designed to compensate for input saturation

(35) $$\begin{array}{l} {\dot{\lambda }}_{1}=-c_{1}\lambda_{1}+\lambda_{2},\\ {\dot{\lambda }}_{2}=-c_{2}\lambda_{2}+D_{a}\Delta \tau_{a}, \end{array}$$

where

$$\lambda_{1}=\left[\begin{array}{c} \lambda_{11}\\ \lambda_{12}\\ \lambda_{13} \end{array}\right],\lambda_{2}=\left[\begin{array}{c} \lambda_{21}\\ \lambda_{22}\\ \lambda_{23} \end{array}\right],c_{1}=\left[\begin{array}{ccc} c_{11} & 0 & 0\\ 0 & c_{12} & 0\\ 0 & 0 & c_{13} \end{array}\right],c_{2}=\left[\begin{array}{ccc} c_{21} & 0 & 0\\ 0 & c_{22} & 0\\ 0 & 0 & c_{23} \end{array}\right],c_{i,j}>0,i=1,2,j=1,2,3.$$

The sliding mode functions are defined as follows

(36) $$\begin{array}{l} z_{1}=\int_{0}^{t}\left(v_{d}-v_{a}\right)d\tau +\lambda_{1},\\ z_{2}=c_{3}z_{1}+{\dot{z}}_{1},\\ z_{3}=c_{4}z_{1}+z_{2}, \end{array}$$

where $v_{d}={\left[\begin{array}{ccc} u_{d} & q_{d} & r_{d} \end{array}\right]}^{T}$. The Lyapunov function is defined as follows:

(37) $$V_{2}=\frac{1}{2}\left(z_{1}^{T}z_{1}+z_{3}^{T}z_{3}\right)$$

The control law is defined as follows:

(38) $$\tau_{a}=D_{a}^{-1}\left(c_{4}{\dot{z}}_{1}+c_{3}{\dot{z}}_{1}+{\dot{v}}_{d}-C_{a}v_{a}-G_{a}-c_{1}\left(-c_{1}\lambda_{1}+\lambda_{2}\right)-c_{2}\lambda_{2}+\frac{1}{2\gamma^{2}}z_{3}+\frac{1}{2}z_{3}+c_{5}z_{3}\right),$$

where

$$c_{3}=\left[\begin{array}{ccc} c_{31} & 0 & 0\\ 0 & c_{32} & 0\\ 0 & 0 & c_{33} \end{array}\right],c_{4}=\left[\begin{array}{ccc} c_{41} & 0 & 0\\ 0 & c_{42} & 0\\ 0 & 0 & c_{43} \end{array}\right],c_{5}=\left[\begin{array}{ccc} c_{51} & 0 & 0\\ 0 & c_{52} & 0\\ 0 & 0 & c_{53} \end{array}\right],c_{i,j}>0,i=3,4,5,j=1,2,3.$$

Then, the derivative of $V_{2}$ yields

$$\begin{array}{ll} {\dot{V}}_{2} & =z_{1}^{T}{\dot{z}}_{1}+z_{3}^{T}{\dot{z}}_{3},\\ & =z_{1}^{T}\left(z_{2}-c_{3}z_{1}\right)+z_{3}^{T}{\dot{z}}_{3},\\ & =-z_{1}^{T}c_{3}z_{1}+z_{1}^{T}z_{2}+z_{3}^{T}\left(c_{4}{\dot{z}}_{1}+{\dot{z}}_{2}\right),\\ & =-z_{1}^{T}c_{3}z_{1}+z_{1}^{T}z_{2}+z_{3}^{T}\left[c_{4}{\dot{z}}_{1}+c_{3}{\dot{z}}_{1}+{\dot{v}}_{d}-{\dot{v}}_{a}+{\ddot{\lambda }}_{1}\right],\\ & =-z_{1}^{T}c_{3}z_{1}+z_{1}^{T}z_{2}+z_{3}^{T}\left[c_{4}{\dot{z}}_{1}+c_{3}{\dot{z}}_{1}+{\dot{v}}_{d}-\left(C_{a}v_{a}+G_{a}+D_{a}{\bar{\tau }}_{a}+b_{a}\right)+\left(-c_{1}{\dot{\lambda }}_{1}+{\dot{\lambda }}_{2}\right)\right],\\ & =-z_{1}^{T}c_{3}z_{1}+z_{1}^{T}z_{2}+z_{3}^{T}\left[c_{4}{\dot{z}}_{1}+c_{3}{\dot{z}}_{1}+{\dot{v}}_{d}-\left(C_{a}v_{a}+G_{a}+D_{a}{\bar{\tau }}_{a}+b_{a}\right)-c_{1}\left(-c_{1}\lambda_{1}+\lambda_{2}\right)+\left(-c_{2}\lambda_{2}+D_{a}\Delta \tau_{a}\right)\right],\\ & =-z_{1}^{T}c_{3}z_{1}+z_{1}^{T}z_{2}+z_{3}^{T}\left[c_{4}{\dot{z}}_{1}+c_{3}{\dot{z}}_{1}+{\dot{v}}_{d}-\left(C_{a}v_{a}+G_{a}+D_{a}\tau_{a}+b_{a}\right)-c_{1}\left(-c_{1}\lambda_{1}+\lambda_{2}\right)-c_{2}\lambda_{2}\right],\\ & =-z_{1}^{T}c_{3}z_{1}+z_{1}^{T}z_{2}-z_{3}^{T}c_{5}z_{3}-\left(\frac{1}{2\gamma^{2}}z_{3}^{T}z_{3}+\frac{1}{2}z_{3}^{T}z_{3}+z_{3}^{T}b_{a}\right). \end{array}$$

Since

$$-\left(\frac{1}{2\gamma^{2}}z_{3}^{T}z_{3}+\frac{1}{2}z_{3}^{T}z_{3}+z_{3}^{T}b_{a}\right)-\frac{1}{2}\gamma^{2}{\left\|b_{a}\right\|}^{2}+\frac{1}{2}{\left\|z_{3}\right\|}^{2}=-\frac{1}{2}{\left\|\frac{1}{\gamma }z_{3}+\gamma b_{a}\right\|}^{2}\leq 0,$$

then, the derivative of $V_{2}$ yields

$${\dot{V}}_{2}\leq -\left[\begin{array}{cc} z_{1}^{T} & z_{2}^{T} \end{array}\right]Q_{V}\left[\begin{array}{c} z_{1}\\ z_{2} \end{array}\right]+\left(\frac{1}{2}\gamma^{2}{\left\|b_{a}\right\|}^{2}-\frac{1}{2}{\left\|z_{3}\right\|}^{2}\right),$$

where $Q_{V}=\left[\begin{array}{cc} c_{3}+c_{5}c_{4}^{2} & c_{5}c_{4}-\frac{1}{2}I_{3}\\ c_{5}c_{4}-\frac{1}{2}I_{3} & c_{5} \end{array}\right]$. Set the parameters $c_{3},c_{4},c_{5}$ to make $\left|Q_{V}\right|>0$, then

$${\dot{V}}_{2}\leq \frac{1}{2}\gamma^{2}{\left\|b_{a}\right\|}^{2}-\frac{1}{2}{\left\|z_{3}\right\|}^{2}.$$

As long as the conditions $\left\|z_{3}\right\|\geq \gamma \left\|b_{a}\right\|$ are satisfied, then ${\dot{V}}_{2}\leq 0$. Consequently, the system is uniformly ultimately bounded can be guaranteed.

### 3.3. Stability Analysis of Sway and Heave

The dynamic equations of the lateral and vertical velocities can be rewritten as

(39) $$\begin{array}{l} \dot{v}=\frac{d_{22}}{m_{22}}\left(v+\frac{b_{v}-m_{11}ur}{d_{22}}\right),\\ \dot{w}=\frac{d_{33}}{m_{33}}\left(w+\frac{b_{w}+m_{11}uq}{d_{33}}\right). \end{array}$$

The Lyapunov function is defined:

(40) $$V_{3}=\frac{1}{2}\left(m_{22}v^{2}+m_{33}w^{2}\right).$$

Since the conditions $d_{22}<0$ and $d_{33}<0$ are satisfied, the derivative of $V_{3}$ yields

$${\dot{V}}_{3}=d_{22}\left(v^{2}+\frac{b_{v}-m_{11}ur}{d_{22}}v\right)+d_{33}\left(w^{2}+\frac{b_{w}+m_{11}uq}{d_{33}}w\right)\leq d_{22}\left(v^{2}-\left|\frac{b_{v}-m_{11}ur}{d_{22}}\right|\left|v\right|\right)+d_{33}\left(w^{2}-\left|\frac{b_{w}+m_{11}uq}{d_{33}}\right|\left|w\right|\right).$$

As long as the conditions $\left|v\right|\geq \left|\frac{b_{v}-m_{11}ur}{d_{22}}\right|,\left|w\right|\geq \left|\frac{b_{w}+m_{11}uq}{d_{33}}\right|$ are satisfied, then ${\dot{V}}_{3}\leq 0$. Consequently, *v* and *w* are passive-bounded, and uniform ultimate bounding can be concluded [41].

## 4. The Results and Analysis of the Simulation Experiment

In order to verify the performance of the controller, the simulation experiment is carried out. The AUV used in the simulation is the REMUS 100 [42]. The desired path is generated based on a series of waypoints which are displayed in Table 1. The initial position, orientation, and velocity of the AUV are zero. The expected forward speed is $u_{d}=1$ $m.s^{-1}$. The radius of turning circle $R_{k}$ is 10 m. In the simulation, three different control methods are used for comparison. The first and the second methods are traditional control methods based on LOS guidance law. The third method is the improved control method based on MPC. Because the PID controller is simple and does not depend on the system model, it is the most widely used control method in various control fields. In the first control method (LOS+PID), the kinematics controller uses LOS guidance law (11), and the dynamics controller uses the PID controller as follows

(41) $$\begin{array}{l} n_{p}=k_{{}_{p}}^{p}\left(u-u_{d}\right)+k_{{}_{i}}^{p}\int_{0}^{t}\left(u-u_{d}\right)d\tau +k_{{}_{d}}^{p}\dot{u},\\ \delta_{s}=k_{{}_{p}}^{s}\left(\theta_{e}-\theta_{ed}\right)+k_{{}_{i}}^{s}\int_{0}^{t}\left(\theta_{e}-\theta_{ed}\right)d\tau +k_{{}_{d}}^{s}\left({\dot{\theta }}_{e}-{\dot{\theta }}_{ed}\right),\\ \delta_{r}=k_{{}_{p}}^{r}\left(\psi_{e}-\psi_{ed}\right)+k_{{}_{i}}^{r}\int_{0}^{t}\left(\psi_{e}-\psi_{ed}\right)d\tau +k_{{}_{d}}^{r}\left({\dot{\psi }}_{e}-{\dot{\psi }}_{ed}\right). \end{array}$$

The other two methods are proposed in this paper. In the second control method (LOS+SMC), the kinematics and the dynamics controller uses LOS and SMC for path following and obstacle avoidance. In the third control method (MPC+SMC), the kinematics and dynamics controller uses MPC and SMC for path following and obstacle avoidance. The main parameters of the controller are displayed in Table 2. The parameters of three controllers are obtained after tuning. The principle of parameter tuning is to make the AUV converge to the desired path as quickly as possible without overshooting. Two obstacles with a radius of 5 m are set, and their center coordinates in the fixed coordinate system are [72 m, 0 m, 28 m]*^T^* and [136 m, 0 m, 28 m]*^T^*. In the simulation, the hydrodynamic parameters are increased by 20% from 0 s–150 s and decreased by 20% from 250 s–430 s, which can verify the robustness of the controller.

**Figure 3:**
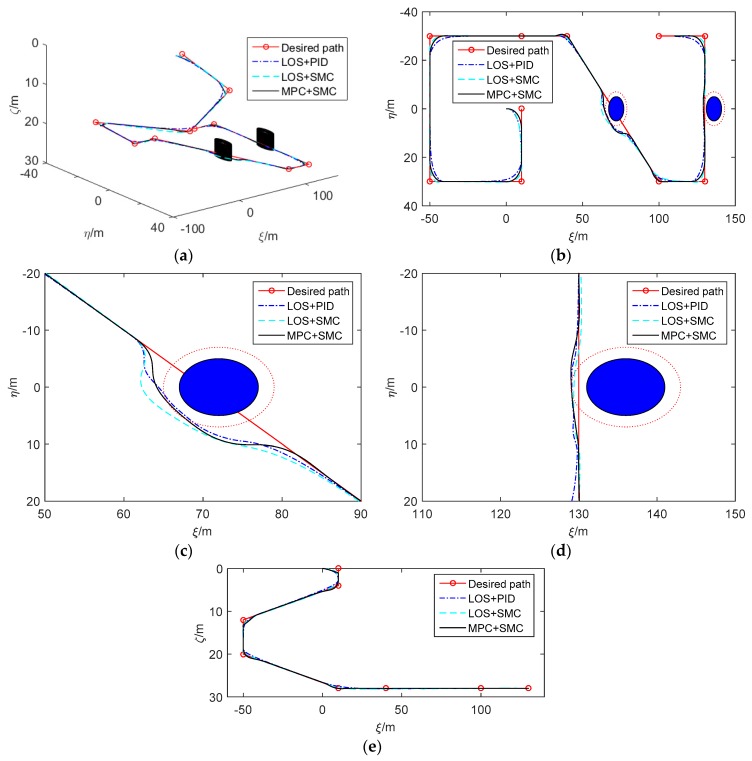
Simulation results of path following and obstacle avoidance. (**a**) The simulation results in 3D. (**b**) The simulation results in the horizontal plane. (**c**) Partial enlarged view of the simulation results (obstacle avoidance 1). (**d**) Partial enlarged view of the simulation results (obstacle avoidance 2). (**e**) The path following is shown in the vertical plane.

**Figure 4:**
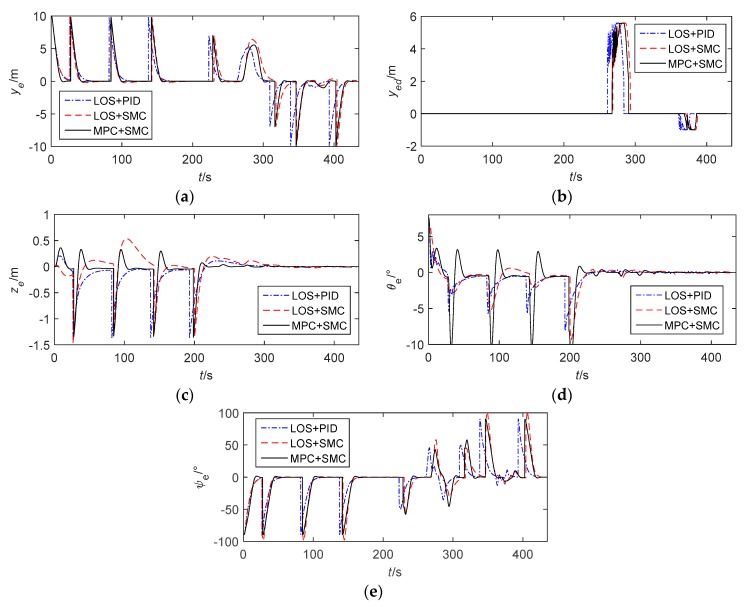
Path following errors. (**a**) The position error (lateral). (**b**) The obstacle avoidance penalty item (lateral). (**c**) The position error (vertical). (**d**) The orientation error (pitch). (**e**) The orientation error (yaw).

**Figure 5:**
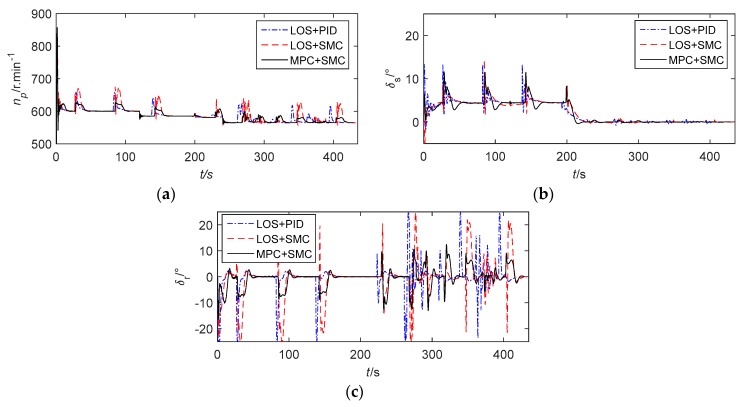
The actual control input variables. (**a**) The speed of the propeller. (**b**) The sternplane rudder angle. (**c**) The vertical rudder angle.

**Figure 6:**
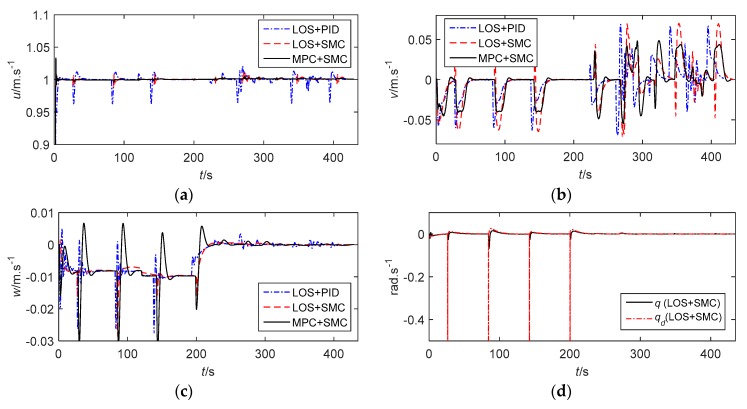

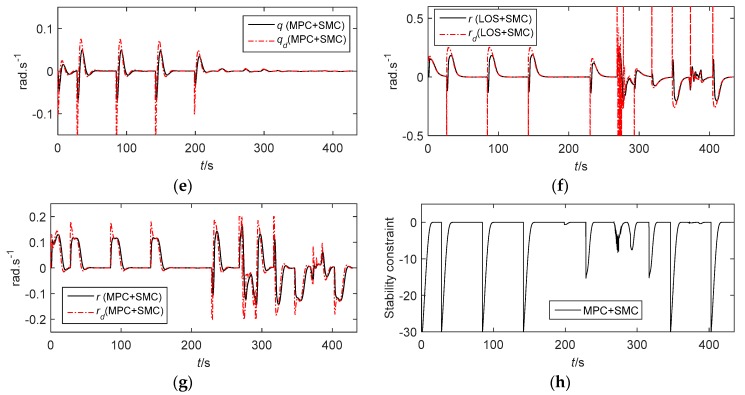
The velocities of the AUV and the stability constraints of MPC. (**a**) Displacement velocity of the AUV (surge). (**b**) Displacement velocity of the AUV (sway). (**c**) Displacement velocity of the AUV (heave). (**d**) Angular velocity of the AUV with LOS+SMC (pitch). (**e**) Angular velocity of the AUV with MPC+SMC (pitch). (**f**) Angular velocity of the AUV with LOS+SMC (yaw). (**g**) Angular velocity of the AUV with MPC+SMC (yaw). (**h**) The stability constraints of MPC.

The simulation results are displayed in Figure 3, Figure 4, Figure 5 and Figure 6. Figure 3a shows the simulation results which are displayed in 3D. Figure 3b is the path following result projection in the horizontal plane. Figure 3c–d are partial zooms of horizontal obstacle avoidance. Figure 3e is the path following results projection in the vertical plane. The simulation results show that the tracking performance of the three control methods is nearly ideal. However, LOS+SMC and MPC+SMC work slightly better than LOS+PID when path switching. In addition, MPC+SMC can converge to the expected lateral error faster and more accurately so as to better achieve obstacle avoidance.

Figure 4 illustrates the path following errors. Obviously, all tracking errors converge to zero on each straight path. We can see that in order for the AUV to avoid obstacles at a safe distance, obstacle avoidance from the right is achieved by setting the expected lateral error to about 5 m when the AUV passes the first obstacle, and obstacle avoidance from the left is achieved by setting the expected lateral error to about −1 m when the AUV passes the second obstacle. When obstacle avoidance is completed, the AUV returns to the desired path by setting the expected lateral error to zero. At the same time, it can be seen that MPC+SMC controls the depth error more smoothly than the other two methods. Figure 5 shows the actual control inputs of the AUV. It is clearly visible that control signals have regular chattering. The chattering is caused by the path switching, because the path is not smooth at each waypoint. The maximum amplitude of the rudder angle is set to ±25°. When LOS+PID and LOS+SMC are adopted, the vertical rudder angle is prone to saturation when the path is switched. When the improved control method (MPC+SMC) is adopted, there is no saturation of the rudder angle, because the constraints on angular velocity are taken into account by the MPC algorithm.

In addition, the MSE of vertical rudder angle is reduced by about 40% compared with that of the traditional control methods, which is more conducive to the system stability and saves more energy. Figure 6 shows the velocities of the AUV and the stability constraints of MPC. The forward speed can converge to the expected value. However, the performance of LOS+SMC and MPC+SMC is better than that of LOS+PID because the forward speed is more stable. At the same time, it can be seen clearly that although the AUV generates certain lateral and vertical velocities due to the coupling between motions, both lateral and vertical velocities are small and convergent. When LOS+SMC is adopted, the expected angular velocities are too large during path switching, which is the reason for the saturation of the rudder angle. The angular velocity of the AUV can not only converge to the expected value but is also within the constraint range with the MPC+SMC method. Figure 6h illustrates the stability constraints of MPC.

## 5. Conclusions

This paper studies two important control tasks of the underactuated AUV, that is, path following and obstacle avoidance. The proposed control method improves the performance of the control system through the following measures. Firstly, in the kinematics controller, the optimal expected angular velocity is designed by using MPC, and the penalty item for obstacle avoidance is designed by using the obstacle information detected by onboard sensors, which can not only reduce the MSE and saturation of the rudder angle but also realize real-time obstacle avoidance. Secondly, the stability constraint conditions are designed, which can guarantee the stability of MPC. The prediction model in MPC adopts the linear time-varying model, which can effectively reduce the computation of the controller. The simulation is implemented in Matlab and Equation (29) is solved by the QP algorithm on a PC (CPU: Intel i5-3230M, 2.6GHz; RAM: 4GB). The average time used to calculate MPC guidance law is within 10 ms. Therefore, the controller can meet the real-time requirement of path following. Because the kinematics level is not affected by the dynamics model uncertainties, the accuracy of MPC can be guaranteed. Thirdly, in the dynamics controller, the saturation of the control input is considered. The actual control signal is designed by SMC to realize the velocity control, which can not only overcome the uncertainty of dynamics model but also ensure the stability of the system. A comparison of the improved control method (MPC+SMC) and the traditional control methods based on LOS guidance law shows that MPC+SMC can yield a superior performance. The MPC+SMC not only better realize the path following and obstacle avoidance but also reduce the MSE and saturation of the rudder angle effectively. Therefore, the MPC+SMC can be more conducive to the stability of the system and can save energy. Fourthly, the obstacle avoidance method designed in this paper is easier to calculate than other obstacle avoidance methods based on path replanning, and therefore it is more convenient for practical application. How to extend the method to solve the problem of curve path tracking and dynamic obstacle avoidance will be considered in the future.

## Figures and Tables

**Table 1:** Waypoints.

Waypoints	1	2	3	4	5	6	7	8	9	10
ξ/m	10	10	−50	−50	10	40	100	130	130	100
η/m	0	30	30	−30	−30	−30	30	30	−30	−30
ζ/m	0	4	12	20	28	28	28	28	28	28

**Table 2:** Parameters of the controller.

MPC	MPC	LOS+PID	LOS+PID	SMC	SMC
T=0.1	$T_{1}=1$	$\Delta_{\theta }=10$	$\Delta_{\psi }=10$	$c_{11}=1$	$c_{12}=1$
$T_{2}=1$	$Q_{11}=2$	$k_{p}^{p}=20$	$k_{i}^{p}=3$	$c_{13}=1$	$c_{21}=1$
$Q_{22}=2$	$Q_{33}=2$	$k_{d}^{p}=8$	$k_{p}^{s}=2$	$c_{22}=1$	$c_{23}=1$
$Q_{44}=2$	$Q_{55}=1$	$k_{i}^{s}=0.1$	$k_{d}^{s}=2$	$c_{31}=0.1$	$c_{32}=0.1$
$Q_{66}=1$	$R_{11}=2$	$k_{p}^{r}=2$	$k_{i}^{r}=0.1$	$c_{33}=0.1$	$c_{41}=0.5$
$R_{22}=2$	$N_{P}=8$	$k_{d}^{r}=2$	$k_{q}=0.2$	$c_{42}=0.5$	$c_{43}=0.5$
$u_{max}={\left[0.15,0.2\right]}^{T}$	$N_{c}=3$	$k_{r}=0.2$	$R_{k}=10$	$c_{51}=1$	$c_{52}=1$
$u_{min}={\left[-0.15,-0.2\right]}^{T}$		$u_{d}=1$	$y_{e}^{s}=2$	$c_{53}=1$	γ=0.2
